# Supplementary material for: The Fertility Indicator Equation Using Serum Progesterone and Urinary Pregnanediol-3-Glucuronide for Assessment of Ovulatory to Luteal Phase Transition
Source: Medicina (Kaunas). 2021 Feb 3;57(2):134. doi: 10.3390/medicina57020134 (PMC7913371; doi:10.3390/medicina57020134)
Supplement: Supplementary file 1 [file medicina-57-00134-s001.pdf]

Supplementary Data. Computation of values for Fertility Indicator Equation (FIE) using day-specific serum progesterone (P) levels and day-specific urinary pregnanediol-3-glucuronide (PDG).

The serum P levels are from: Stricker et al., (18), mean, 5th percentile(PCTL), and 95th PCTL levels and Roos et al., (19), median, 10th and 90th PCTL levels.

The urinary PDG levels, not adjusted for urinary concentration or excretion rate, are from: Johnson et al., (20), μg/ml, median, 10th PCTL, and 90th PCTL levels, and Alliende et al., (supplementary data below, also see ref 14), μmol/L, mean, median, 5th, 10th, 90th, and 95th PCTL.

The levels are indexed to the day of ovulation, Day 0. The day of ovulation was determined by ultrasonogram for Roos et al., and Johnson et al. For Stricker et al., the day ovulation was considered the day after the serum LH peak. The day of ovulation for Alliende et al., was considered the day after the urinary LH surge (supplement 1 in ref 14).

The Delta and FIE values for day, D, in a cycle were computed as described by Usala and Trindade (17). $Delta_D$ for P or PDG levels is calculated for day, D, by: [(P or PDG on day, D) – (P or PDG on day, D-1)]/(P or PDG on day, D-1) and $Delta_{D-1}$ for day, D-1, by: [(P or PDG on Day, D-1) – (P or PDG on day, D-2)]/(P or PDG on day, D-2). The magnitude of FIE on day, D, (that is, magnitude of FIE(D)) is calculated by: (($Delta_D$ X ($Delta_{D-1}$ X 100). The sign of FIE(D) is +, -, or indeterminate (written as ind) and is assigned as follows: +$Delta_D$ X +$Delta_{D-1}$ is +; -$Delta_D$ X -$Delta_{D-1}$ is -; and -$Delta_D$ X +$Delta_{D-1}$ or +$Delta_D$ X -$Delta_{D-1}$ is ind. In the attached work above, an indeterminate value for FIE is left blank, since it provides no information as to the change in P or PDG.

Note the conversion for PDG units: PDG 5μg/ml = PDG 5mg/L => PDG 5mg/L x 3.12 = PDG μmol/L

Day of Cycle	Stricker P mean nmol/L	Delta Stricker mean	FIE Stricker mean	Stricker P 5th PCTL nmol/L	Delta Stricker 5th PCTL	FIE Stricker 5th PCTL	Stricker P 95th PCTL nmol/L	Delta Stricker 95th PCTL	FIE Stricker 95th PCTL
-16	1.66			0.32			3.75		
-15	1.27	-0.2349		0.32	0.0000		2.73	-0.2720	
-14	1.02	-0.1969	-4.6248	0.52	0.6250	0	1.59	-0.4176	-11.3582
-13	0.82	-0.1961	-3.8598	0.32	-0.3846	ind24.0385	1.65	0.0377	ind1.5758

-12	0.74	-0.0976	-1.913	0.32	0.0000	ind0	1.3	-0.2121	ind0.8005
-11	0.94	0.2703	ind2.6368	0.32	0.0000	0	2.32	0.7846	ind16.6434
-10	0.72	-0.2340	ind6.3255	0.32	0.0000	0	1.29	-0.4440	ind34.8342
-9	0.67	-0.0694	-1.6253	0.32	0.0000	0	1.61	0.2481	ind1.11.0131
-8	0.73	0.0896	ind0.6219	0.32	0.0000	0	1.3	-0.1925	ind4.7763
-7	0.51	-0.3014	ind2.6988	0.32	0.0000	0	1	-0.2308	-4.4434
-6	0.57	0.1176	ind3.5455	0.32	0.0000	0	1.02	0.0200	ind0.4615
-5	0.59	0.0351	0.4128	0.32	0.0000	0	0.95	-0.0686	ind0.1373
-4	0.51	-0.1356	ind0.4758	0.32	0.0000	0	0.97	0.0211	ind0.1445
-3	0.59	0.1569	ind2.1270	0.32	0.0000	0	1.27	0.3093	0.6511
-2	1.02	0.7288	11.4324	0.62	0.9375	0	1.62	0.2756	8.5234
-1	2.66	1.6078	117.1818	1.24	1.0000	93.75	4.13	1.5494	42.6995
0	5.02	0.8872	142.6507	2.21	0.7823	78.2258	8.95	1.1671	180.82
1	12.13	1.4163	125.6598	4.74	1.1448	89.5526	18.67	1.0860	126.7477
2	20.84	0.7181	101.7005	11.34	1.3924	159.4020	29.05	0.5560	60.3804
3	29.74	0.4271	30.6655	11.38	0.0035	0.4911	45.51	0.5666	31.5019
4	36.08	0.2132	9.1042	23.72	1.0844	0.3825	46.46	0.0209	1.1828
5	36.52	0.0122	0.2600	22.36	-0.0573	ind6.2172	48.18	0.0370	0.0773
6	40.32	0.1041	0.1269	25.98	0.1619	ind0.9282	54.17	0.1243	0.4603
7	39.65	-0.0166	ind0.1729	25.06	-0.0354	ind0.5733	60.39	0.1148	1.4276
8	33.76	-0.1485	-0.2468	18.25	-0.2717	-0.9623	46.65	-0.2275	ind2.6125
9	34.11	0.0104	ind0.1540	17.74	-0.0279	-0.7594	55.94	0.1991	ind4.5309
10	18.24	-0.4653	ind0.4823	6.74	-0.6201	-1.7327	38.21	-0.3169	ind6.3118
11	16.9	-0.0735	-3.418	3.67	-0.4555	-28.2434	41.58	0.0882	ind2.7954
12	10.73	-0.3651	-2.6821	2.15	-0.4142	-18.8649	33.15	-0.2027	ind1.7881
13	9.5	-0.1146	-4.1851	1.91	-0.1116	-4.6232	33.39	0.0072	ind0.1468

Day of Cycle	Roos P median nmol/L	Delta Roos median	FIE Roos median	Roos P 10th PCTL nmol/L	Delta Roos 10th PCTL	FIE Roos 10th PCTL	Roos P 90th PCTL nmol/L	Delta Roos 90th PCTL	FIE Roos 90th PCTL
-16	2.4			1.8			2.7		
-15	1.9	-0.2083			-1.0000	ind			
-14	1	-0.4737	-9.8684	0.8	#DIV/0!	ind	2.4	#DIV/0!	ind
-13	1.6	0.6000	ind28.421 1	0.9	0.1250	ind	1.9	-0.2083	ind
-12	2.1	0.3125	18.75	1.7	0.8889	11.1111	2.2	0.1579	ind3.2895
-11	1.5	-0.2857	ind8.9285	0.3	-0.8235	ind73.2026	2.2	0.0000	0
-10	1.5	0.0000	ind0	0.5	0.6667	ind54.9019	2	-0.0909	ind0
-9	1.4	-0.0667	ind0	0.3	-0.4000	ind26.6667	2.5	0.2500	ind2.2727
-8	1	-0.2857	-1.9047	0.4	0.3333	ind13.3333	2.6	0.0400	1
-7	0.8	-0.2000	-5.7142	0.5	0.2500	8.3333	1.6	-0.3846	ind1.5384
-6	1.3	0.6250	ind12.5	0.5	0.0000	0	2.6	0.6250	ind24.0385
-5	1.4	0.0769	4.8076	0.5	0.0000	0	2.1	-0.1923	ind12.0192
-4	0.9	-0.3571	ind2.7472	0.4	-0.2000	ind0	2.4	0.1429	ind2.7472
-3	1.3	0.4444	ind15.873	0.6	0.5000	ind10.00	2.3	-0.0417	ind0.5952
-2	1.9	0.4615	20.5128	0.8	0.3333	16.6667	3.8	0.6522	ind2.7173
-1	3.1	0.6316	29.1498	2	1.5000	50.0000	5	0.3158	20.5950
0	4.6	0.4839	30.5603	2.9	0.4500	67.5000	9.7	0.9400	29.6842
1	10.3	1.2391	59.9579	7.2	1.4828	66.7241	15.6	0.6082	57.1753
2	20.7	1.0097	125.1161	8	0.1111	16.4751	32.9	1.1090	67.4531
3	22.7	0.0966	9.7556		-1.0000	ind			ind
4	33.4	0.4714	4.5543		#DIV/0!	ind		#DIV/0!	ind
5	30.8	-0.0778	ind3.6693		#DIV/0!	ind		#DIV/0!	ind

6	36.3	0.1786	ind1.3900	20.2	#DIV/0!	ind	63.9	#DIV/0!	ind
7	32	-0.1185	ind2.1153	21.8	0.0792	ind	63.1	-0.0125	ind
8	32.7	0.0219	ind0.2591	21.4	-0.0183	ind0.1453	56.7	-0.1014	-0.127
9	33.1	0.0122	0.0267	17.1	-0.2009	-0.3686	63.4	0.1182	ind1.198
10	21.3	-0.3565	ind0.4360	11	-0.3567	-7.1678	29.7	-0.5315	ind6.2811
11	17.1	-0.1972	-7.0294	9.8	-0.1091	-3.8915	24.4	-0.1785	-9.4855
12					-1.0000	ind			
13					#DIV/0!	ind			

Day of Cycle	Johnson PDG median mcg/ml	Delta Johnson median	FIE Johnson median	Johnson PDG 10th PCTL mcg/ml	Delta Johnson 10th PCTL	FIE Johnson 10th PCTL	Johnson PDG 90th PCTL mcg/ml	Delta Johnson 90th PCTL	FIE Johnson 90th PCTL
-16	3.4			1			5.6		
-15	3.4	0.0000		0.8	-0.2000		10.4	0.8571	
-14	2.9	-0.1471	ind0	0.8	0.0000	ind0	5.5	-0.4712	ind40.3846
-13	2.8	-0.0345	-0.5071	0.9	0.1250	0	5.4	-0.0182	-0.8566
-12	2.1	-0.2500	-0.8621	0.8	-0.1111	ind1.3889	6.1	0.1296	ind0.2357
-11	2.2	0.0476	ind1.1905	1	0.2500	ind2.7778	4.9	-0.1967	ind2.5501
-10	2.1	-0.0455	ind0.2165	1	0.0000	ind0	4.9	0.0000	ind0
-9	2.1	0.0000	ind0	0.9	-0.1000	ind0	4.2	-0.1429	ind0
-8	2.2	0.0476	0	0.9	0.0000	ind0	4.5	0.0714	ind1.0204
-7	1.8	-0.1818	ind0.8658	0.7	-0.2222	ind0	3.8	-0.1556	ind1.1111
-6	1.8	0.0000	ind0.0000	0.8	0.1429	ind3.1746	4.3	0.1316	ind2.0468
-5	1.9	0.0556	0	0.9	0.1250	1.7857	3.9	-0.0930	ind1.2240
-4	1.9	0.0000	0	0.9	0.0000	0	3.1	-0.2051	-1.9082
-3	2	0.0526	0	1	0.1111	0	4.1	0.3226	ind6.6170
-2	2.6	0.3000	1.5789	1	0.0000	0	4.5	0.0976	3.1471
-1	2.3	-0.1154	ind3.4615	1.1	0.1000	0	5.5	0.2222	2.1680

0	3.4	0.4783	ind5.5184	1.6	0.4545	4.5455	7.7	0.4000	8.8889
1	4.3	0.2647	12.6598	1.7	0.0625	2.8409	8.5	0.1039	4.1558
2	6.5	0.5116	13.5431	2.7	0.5882	3.6765	14.7	0.7294	7.5783
3	9	0.3846	19.6780	4	0.4815	28.3224	25.8	0.7551	55.0780
4	13.5	0.5000	19.2308	4.6	0.1500	7.2222	32.1	0.2442	18.4385
5	17.1	0.2667	13.3333	5.3	0.1522	2.2826	53.6	0.6698	16.3551
6	18.1	0.0585	1.5595	5.7	0.0755	1.1485	59	0.1007	6.7478
7	18.7	0.0331	0.1939	8.2	0.4386	3.3102	41	-0.3051	ind3.0736
8	18.6	-0.0053	ind0.0177	9.7	0.1829	8.0231	35.9	-0.1244	-3.795
9	19.9	0.0699	ind0.0374	6.2	-0.3608	ind6.6005	47.3	0.3175	ind3.9500
10	13.1	-0.3417	ind2.3883	6.5	0.0484	ind1.7459	36.3	-0.2326	ind7.3849
11	11	-0.1603	-5.4778	4.4	-0.3231	ind1.5633	29.9	-0.1763	-4.1002
12	9.6	-0.1273	-2.0402	4.5	0.0227	ind0.7343	19.9	-0.3344	-5.8966
13	8.8	-0.0833	-1.0606	3.8	-0.1556	ind0.3535	18.6	-0.0653	-2.1848

Day of Cycle	Alliende PDG mean μmol/L	Delta Alliende mean	FIE Alliende mean	Alliende PDG 5th PCTL μmol/L	Delta Alliende 5th PCTL	FIE Alliende 5th PCTL	Alliende PDG 95th PCTL μmol/L	Delta Alliende 95th PCTL	FIE Alliende 95th PCTL
-16	1.4670			0.566			6.04		
-15	1.0553	-0.2806		0.317	-0.4399		2.627	-0.5651	
-14	1.4665	0.3896	ind10.9334	0.44	0.3880	ind17.0689	4.4105	0.6789	ind38.3630
-13	1.4034	-0.0430	ind1.6768	0.293	-0.3341	ind12.9631	3.552	-0.1946	ind13.2149
-12	1.3254	-0.0556	-0.2393	0.3065	0.0461	ind1.5393	3.882	0.0929	ind1.8084
-11	1.4634	0.1041	ind0.5790	0.322	0.0506	0.233	4.005	0.0317	0.2944
-10	1.3062	-0.1074	ind1.1181	0.262	-0.1863	ind0.9423	3.5785	-0.1065	ind0.3374
-9	1.2103	-0.0735	-0.7889	0.29	0.1069	ind1.9914	2.71	-0.2427	-2.5846
-8	1.2600	0.0411	ind0.3019	0.33	0.1379	1.4741	3.1665	0.1685	ind4.0883

-7	1.1928	-0.0533	ind0.2191	0.187	-0.4333	ind5.9770	3.463	0.0936	1.5773
-6	1.1336	-0.0497	-0.2648	0.23	0.2299	ind9.9643	2.578	-0.2556	ind2.3930
-5	1.0003	-0.1176	-0.5841	0.171	-0.2565	ind5.8986	2.196	-0.1482	-3.7868
-4	1.0591	0.0588	ind0.6920	0.17	-0.0058	-0.15	2.39	0.0883	ind1.3090
-3	1.1368	0.0734	0.4316	0.172	0.0118	ind0.0069	2.759	0.1544	1.3639
-2	1.2072	0.0619	0.4544	0.241	0.4012	0.4720	3.045	0.1037	1.6005
-1	1.6792	0.3910	2.4221	0.416	0.7261	29.1301	3.969	0.3034	3.1456
0	2.0194	0.2026	7.9195	0.441	0.0601	4.3638	5.184	0.3061	9.2892
1	2.5074	0.2417	4.8956	0.571	0.2948	1.7715	6.433	0.2409	7.3755
2	4.1171	0.6419	15.5155	0.831	0.4553	13.4228	9.738	0.5138	12.3781
3	6.2057	0.5073	32.5666	1.836	1.2094	55.0684	16.382	0.6823	35.0524
4	8.1383	0.3114	15.7993	1.952	0.0632	7.6410	23.966	0.4629	31.5858
5	8.6022	0.0570	1.7751						
6	9.3392	0.0857	0.4884						
7	11.2996	0.2099	1.7985						
8	9.1410	-0.1910	ind4.0099						
9	9.7823	0.0702	ind1.3401						
10									

Day of Cycle	Alliende PDG median µmol/L	Delta Alliende median	FIE Alliende median	Alliende PDG 10th PCTL µmol/L	Delta Alliende 10th PCTL	FIE Alliende 10th PCTL	Alliende PDG 90th PCTL µmol/L	Delta Alliende 90th PCTL	FIE Alliende 90th PCTL

-16	1.05			0.608			2.271		
-15	0.86	-0.1810		0.439	-0.2780		1.695	-0.254	
-14	1.05	0.2209	ind3.9978	0.464	0.0569	ind1.5829	2.774	0.637	ind16.146
-13	1.115	0.0619	1.3677	0.457	-0.0151	ind0.0859	2.953	0.065	4.108
-12	0.9	-0.1928	ind1.1937	0.434	-0.0503	-0.0759	2.876	-0.026	ind0.168
-11	1.01	0.1222	ind2.3568	0.456	0.0507	ind0.2551	3.402	0.183	ind0.477
-10	0.98	-0.0297	ind0.3630	0.336	-0.2632	ind1.3340	2.656	-0.219	ind4.011
-9	0.95	-0.0306	-0.0909	0.365	0.0863	ind2.2713	2.27	-0.145	-3.187
-8	1.03	0.0842	ind0.2578	0.524	0.4356	3.7598	2.414	0.063	ind0.922
-7	0.86	-0.1650	ind1.3899	0.27	-0.4847	ind21.1158	2.465	0.021	0.134
-6	0.945	0.0988	ind1.6313	0.389	0.4407	ind21.3642	2.118	-0.141	ind0.297
-5	0.88	-0.0688	ind0.6798	0.3	-0.2288	ind10.0838	1.919	-0.094	-1.323
-4	0.885	0.0057	ind0.0391	0.321	0.0700	ind1.6015	1.832	-0.045	-0.426
-3	1.045	0.1808	0.1027	0.279	-0.1308	ind0.9159	2.186	0.193	ind0.876
-2	1.075	0.0287	0.5190	0.348	0.2473	ind3.2359	2.407	0.101	1.954
-1	1.36	0.2651	0.7611	0.651	0.8707	21.5332	3.1	0.288	2.911
0	1.61	0.1838	4.8735	0.48	-0.2627	ind22.8706	4.233	0.365	10.523
1	2.05	0.2733	5.0237	0.851	0.7729	ind20.3024	4.245	0.003	0.104
2	3.08	0.5024	13.7313	1.488	0.7485	57.8552	7.853	0.850	0.241
3	4.95	0.6071	30.5052	2.019	0.3569	26.7117	13.179	0.678	57.644
4	6.775	0.3687	22.3846	2.715	0.3447	12.3017	14.054	0.066	4.503
5	6.97	0.0288	1.0612	3.758	0.3842	13.2430	17.152	0.220	1.464
6	7.435	0.0667	0.1920	3.767	0.0024	0.0920	15.972	-0.069	ind1.517
7	8.76	0.1782	1.1889	3.996	0.0608	0.0146	19.164	0.200	ind1.375
8	7.84	-0.1050	ind1.8716	2.686	-0.3278	ind1.9929	15.842	-0.173	ind3.464
9	7.99	0.0191	ind0.2009	3.208	0.1943	ind6.3710	16.838	0.063	ind1.090
10	6.26	-0.2165	ind0.4143	2.536	-0.2095	ind4.0710	13.05	-0.225	ind1.414
11	4.61	-0.2636	-5.7070	2.076	-0.1814	-3.7996	13.264	0.016	ind0.369
12	3.81	-0.1735	-4.5740	1.268	-0.3892	-7.0598	8.12	-0.388	ind0.636
13	3.26	-0.1444	-2.5051	1.26	-0.0063	-0.2456	6.146	-0.243	-9.428
